# Eco‐immunology and bioinvasion: revisiting the evolution of increased competitive ability hypotheses

**DOI:** 10.1111/eva.12406

**Published:** 2016-07-22

**Authors:** Stéphane Cornet, Carine Brouat, Christophe Diagne, Nathalie Charbonnel

**Affiliations:** ^1^ Centre de Biologie Pour la Gestion des Populations (UMR INRA/IRD/CIRAD/Montpellier SupAgro)IRDMontferrier‐sur‐LezFrance; ^2^ Centre de Biologie Pour la Gestion des Populations (UMR INRA/IRD/CIRAD/Montpellier SupAgro)IRDCampus de Bel‐Air, DakarSénégal; ^3^ Département de Biologie Animale, Faculté des Sciences et TechniquesUniversité Chiekh Anta DiopFann, DakarSénégal; ^4^ Centre de Biologie Pour la Gestion des Populations (UMR INRA/IRD/CIRAD/Montpellier SupAgro)INRAMontferrier‐sur‐LezFrance

**Keywords:** competitive ability, costly immune defences, expansion range, host–parasite coevolution, inflammation, invasive species

## Abstract

Immunity is at the core of major theories related to invasion biology. Among them, the evolution of increased competitive ability (EICA) and EICA‐refined hypotheses have been used as a reference work. They postulate that the release from pathogens often experienced during invasion should favour a reallocation of resources from (costly) immune defences to beneficial life‐history traits associated with invasive potential. We review studies documenting immune changes during animal invasions. We describe the designs and approaches that have been applied and discuss some reasons that prevent drawing generalized conclusions regarding EICA hypotheses. We detail why a better assessment of invasion history and immune costs, including immunopathologies and parasite communities, could improve our understanding of the relationships between immunity and invasion success. Finally, we propose new perspectives to revisit the EICA hypotheses. We first emphasize the neutral and adaptive mechanisms involved in immune changes, as well as timing of the later. Such investigation will help decipher whether immune changes are a consequence of pre‐adaptation, or the result of postintroduction adaptations to invasion front conditions. We next bring attention to new avenues of research that remain unexplored, namely age‐dependent immunity and gut microbiota, potential key factors underlying adaptation to invasion front environment and modulating invasion success.

## 1 Introduction

Despite the importance of biological invasions and their detrimental consequences for biodiversity, public health and economy (Pimentel et al., 2001; Sax, Stachowicz, & Gaines, 2005), the ecological and evolutionary forces explaining why some introduced populations become invasive need to be better understood (Facon et al., 2006; Kolar & Lodge, 2001). Several life‐history traits have been examined because of their potential predictive power of invasion success, such as dispersal or reproduction (Sakai et al., 2001). Although less directly related to population expansion, immunity is a complex physiological function that is at the core of major theories related to invasion success (Dunn et al., 2012; White & Perkins, 2012).

Numerous arguments suggest that ecological conditions influencing immune defences are likely to vary for invasive species throughout their invaded range. For instance, introduced species may lose native parasites (Colautti, Ricciardi, Grigorovich, & MacIsaac, 2004). Based on optimal defence theory, the evolution of increased competitive ability (EICA, Blossey & Nötzold, 1995) hypothesis suggests that such escape from parasites should favour introduced species that decrease investment in immunity and allocate resources to dispersal and reproduction, thereby enhancing their invasive potential (Colautti et al., 2004). However, this hypothesis ignores that invaders are also likely to encounter new parasites in recently invaded areas and during their expansion range (Kelly, Paterson, Townsend, Poulin, & Tompkins, 2009): total immune suppression or reduced immune responses may thus be highly risky and counter‐selected. Immune responses have varying costs in terms of development, maintenance and activation (see Klasing, 2004; Lee, 2006 and references therein). When disproportionate or misdirected, they also trigger collateral damages known as immunopathologies (Graham, Allen, & Read, 2005; Sears, Rohr, Allen, & Martin, 2011; Sorci & Faivre, 2009). Hence, local and/or systemic inflammation incurs high‐energy expenditure and other physiological costs, including immunopathological ones (Ashley, Weil, & Nelson, 2012; Lee & Klasing, 2004; Martin, Hopkins, Mydlarz, & Rohr, 2010), compared with responses mediated by other innate or adaptive effectors (Råberg et al., 2002). Given the trade‐offs between immune pathways mediated by these costs, Lee and Klasing (2004) proposed that successful colonizers should instead be those that dampen the most energetically expensive and/or the least effective immune defences (with regard to the pathogens lost and those newly encountered on the invasion front) to the benefit of less costly and more efficient immune strategies (the EICA‐refined hypothesis). In the case of invading vertebrate species for example, a dampened inflammatory response is expected to favour less costly responses, such as antibody‐mediated immunity (Lee & Klasing, 2004; Martin, Hopkins, et al. 2010).

Lee and Klasing's (2004) EICA‐refined hypothesis represents a cornerstone for eco‐immunological studies dealing with bioinvasions. Testing predictions connecting invasion success and investment in immune defences has, however, proven challenging. In this article, we review the empirical studies documenting immune responses in invasive invertebrate or vertebrate species. We describe and discuss the benefits and the pitfalls of the methodological designs used to test the EICA hypotheses and detail how they have contributed to increase our knowledge of this developing field. We identify several aspects that appear neglected or overlooked in current studies, and we propose new directions to revisit the EICA and EICA‐refined hypotheses. These perspectives should improve our understanding of the relationships between immune strategies and invasion.

## 2 Evidence for immune changes during invasion?

Eco‐immunology in invasion biology is a developing field. A survey of this literature with a focus on empirical studies, irrespective of experimental designs and underlying hypotheses, yielded only 16 studies (Table 1). They include 12 covering vertebrates, but only four on invertebrates. The number of papers reporting studies conducted on the same vertebrate model (e.g. toads, sparrows) puts in perspectives the seemingly under‐representation of invertebrate studies. Moreover, the vertebrate literature is essentially biased towards avian models (Table 1), despite the great number of other vertebrate species reported to be in expansion range (see for instance the “Global Invasive species database”: http://www.issg.org/database/species/search.asps). This point reflects a general bias known in the eco‐immunology literature (Schmid‐Hempel, 2003).

**Table 1:** Studies investigating immune changes in the context of bioinvasion and their input to the evolution of increased competitive ability (EICA) hypothesis

Taxon	Experimental design	Biological system^a^	Immune parameters investigated^b^	Comparison	Support EICA^c^	Reference
Invertebrates						
Insect	Invasive vs. native	*Polistes dominulus* vs. *P. fuscatus* wasps	● Encapsulation response	Inv < Nat	Yes	Wilson‐Rich and Starks (2010)
			● Phenoloxidase activity	Inv < Nat		
Insect	Invasive vs. native	*Drosophila suzukii* vs. *D. melanogaster* flies	● Circulating hemocyte load	Inv > Nat	No	Kacsoh and Schlenke (2012)
Insect	Invasive vs. reference organism	*Ladybird Harmonia axyridis* vs. *Tribolium castaneum*	● Diversity of immune‐related genes	Inv = Ref	No	Vilcinskas et al. (2013)
			● Diversity of antimicrobial peptide genes	Inv > Ref		
Crustacean	Invasive vs. native	*Gammarus roeseli* vs. *G. pulex* gammarids	● Phenoloxidase activity	Inv < Nat	Yes	Cornet et al. (2010)
			● Circulating hemocyte load	Inv = Nat		
Vertebrates						
Bird	Invasive vs. less invasive	House sparrow *Passer domesticus* vs. tree sparrow *P. montanus*	● Metabolic rate following PHA challenge*	Inv < Nat	Yes	Lee et al. (2005)
Bird	Invasive vs. less invasive	House sparrow *P. domesticus* vs. tree sparrow *P. montanus*	● Antibody titres to KLH/SRBC challenge	Inv > Nat	Mixed	Lee et al. (2006)
			● Duration of local inflammation to PHA challenge*	Inv < Nat		
			● Specific T‐cell memory to KLH	Inv > Nat		
Bird	Invasive vs. native	House sparrow *P. domesticus* vs. rufous sparrow *P. ruficintus*	● Baseline haptoglobin level*	Inv < Nat	Yes	Martin, Alam, et al. (2010)
			● Haptoglobin level after CFA challenge*	Inv = Nat		
Bird	Invasive vs. native	House sparrow *P. domesticus* vs. cliff swallow *Petrochelidon pyrrhonota*	● Antibody titre to a vector‐transmitted virus	Inv > Nat	No	Fassbinder‐Orth et al. (2013)
Fish	Expansion vs. native ranges	Rainbow trout *Onchorhynchus mykiss*, brown trout *Salmo trutta*	● *Mhc* allelic diversity	Front = Establ	Yes	Monzon‐Arguello et al. (2014)
			● *Mhc* functional diversity	Front < Establ		
Amphibian	Ongoing expansion range	Cane toad *Rhinella marina*	● Bacteria‐killing ability	High < Low disp	Mixed	Brown and Shine (2014)
			● Phagocytic activity	High < Low disp		
			● Local inflammation to PHA challenge*	High > Low disp		
Amphibian	Ongoing expansion range	Cane toad *Rhinella marina*	● Metabolic rate following LPS challenge*	Front < Establ	Yes	Llewellyn et al. (2012)
Amphibian	Ongoing expansion range	Cane toad *Rhinella marina*	● Bacteria‐killing ability	Front > Establ	No	Brown et al. (2015)
			● Neutrophil concentration	Front > Establ		
			● Phagocytic activity	Front > Establ		
			● Lymphocyte concentration	Front = Establ		
Bird	Ongoing expansion range	House sparrow *P. domesticus*	● Baseline expression of inflammatory TLR‐2 and TLR‐4 genes*	Front > Establ	No	Martin et al. (2014)
Mammal	Ongoing expansion range	Bank vole *Myodes glareolus*	● Number of genic SNPs on 4 immune‐related genes*	Front > Establ	No	White et al. (2013)
Mammal	Ongoing expansion range	Roe deer *Capreolus capreolus*	● *Mhc* genetic diversity	Front < Establ	Yes	Quéméré et al. (2015)
			● Allelic diversity of inflammatory TLR genes (TLR‐2)*	Front = Establ		
Mammal	Both ongoing expansion range and invasive vs. native	Domestic mouse *Mus musculus domesticus*	● Haptoglobin level*	Front > Establ	No	Diagne et al. (in revision)
			● Natural antibodies (HA/HL)	Front > Establ		
		Black rat *Rattus rattus*	● Haptoglobin level*	Front > Establ	No	
			● Natural antibodies (HA/HL)	Front = Establ		

PHA, Phytohemagglutinin; SRBC, sheep red blood cells; KLH, keyhole limpet haemocyanin; CFA, complete Freund's adjuvant; *Mhc,* major histocompatibility complex; SNP, single nucleotide polymorphism; TLR, Toll‐like receptor; HA/HL, haemagglutination–haemolysis; Inv, invasive species; Nat, native species; Front, frontal population; Establ, established population; Disp, dispersers.

a First mentioned species denotes the invasive species and the second one the native species (unless specified).

b Immune parameters involved in/related to the inflammation process are marked by an asterisk.

c Yes/No/Mixed denotes whether the results support the EICA hypothesis with respect to the relationships between immune defences and invasion success. Yes: Invaders or fontal populations have overall lower levels of defences than natives or established populations. No: Invaders or fontal populations have overall higher levels of defences than natives or established populations. Mixed: opposite patterns were found between immune effectors. When several parameters were investigated, and when no change was observed for an effector, the overall score of the study was driven by the parameters that significantly varied between species/populations.

These 16 studies are based on three types of comparison (Table 1): interspecific comparisons between invasives and either native species or less invasive species (nine studies); intraspecific comparisons of populations in their native versus invaded range (one study); and intraspecific comparisons of invasive populations across their expansion range (seven studies). All invertebrate studies use interspecific comparisons, whereas mammal and amphibian models are investigated mainly through the “expansion range” angle. Birds and rodents have been investigated using both interspecific and intraspecific comparisons.

We observe a great diversity of techniques used to assess immune parameters (Table 1). These include measures of humoral and cellular components belonging to both innate and adaptive (in the case of vertebrates) arms of the immune system, as well as examples of inducible immune responses elicited by an experimental immune challenge (see details in Table 1). Note that all studies used wild‐caught animals, which were most likely exposed and infected by parasites, thus levels of defences estimated actually represent a mixture of constitutive and inducible responses. In addition to classical and nonspecific immune assays (e.g. cell count, enzymatic activity; Boughton, Joop, & Armitage, 2011), we noticed a growing interest in and use of diversified and modern immunology techniques applied to wild, nonmodel systems (Pedersen & Babayan, 2011) such as immuno‐genetic/genomic approaches in both invertebrate and vertebrate systems (Martin, Coon, Liebl, & Schrey, 2014; Monzon‐Arguello, de Leaniz, Gajardo, & Consuegra, 2014; Quéméré et al., 2015; Vilcinskas, Mukherjee, & Vogel, 2013; White, Perkins, Heckel, & Searle, 2013).

A differential investment in immune responses between invasive and native species, between expanding and native or anciently established populations, or between high and low dispersers of an invasive species at the invasion front is found in 25 over the 32 immune parameters listed across all studies (Table 1). Over the 16 studies examined, seven provide evidence for an overall significant negative relationship between invasion success and investment in immunity (Cornet, Sorci, & Moret, 2010; Lee, Martin, & Wikelski, 2005; Llewellyn, Thompson, Brown, Phillips, & Shine, 2012; Martin, Alam, Imboma, & Liebl, 2010; Monzon‐Arguello et al., 2014; Quéméré et al., 2015; Wilson‐Rich & Starks, 2010), thereby supporting the EICA hypothesis (Table 1). In contrast, seven other studies show an overall positive relationship between invasion success and investment in immunity (Brown, Phillips, Dubey, & Shine, 2015; Diagne et al., in revision; Fassbinder‐Orth, Barak, & Brown, 2013; Kacsoh & Schlenke, 2012; Martin et al., 2014; Vilcinskas et al., 2013; White et al., 2013). The two remaining studies show mixed results according to the immune parameters considered (Brown & Shine, 2014; Lee, Martin, Hasselquist, Ricklefs, & Wikelski, 2006).

In vertebrates, only five studies allow testing of the EICA‐refined hypothesis, as they concomitantly analyse immune parameters related to the inflammation process in addition to presumptively less costly processes (Table 1). The EICA‐refined predictions are corroborated in two animal models only. First, invasive house sparrows (*Passer domesticus*) in North America express a weaker inflammatory response to local PHA immune challenge (Lee et al., 2005, 2006), but mount stronger humoral antibody‐mediated responses (Lee et al., 2006) than its less invasive congener, the Eurasian tree sparrow (*Passer montanus*). This finding is confirmed by two other studies carried out in different invasion contexts (Fassbinder‐Orth et al., 2013; Martin, Alam, et al. 2010). Second, cane toads, *Rhinella marina*, sampled on the invasion front in tropical Australia have lower metabolic rate following a systemic LPS challenge (i.e. lower inflammatory response), and express higher innate humoral and cellular defences than individuals collected in long‐established populations (Brown et al., 2015; Llewellyn et al., 2012).

In invertebrates, there is no evidence supporting the EICA‐refined hypothesis. Two studies show a downregulation of phenoloxidase (PO) activity in invaders (Table 1). PO is a general defence and nonself recognition system in arthropods that provides immunity against a large range of pathogens. Nonetheless, its activity leads to the production and release of cytotoxic molecules that generate immunopathologic costs (Cornet, Franceschi, Bollache, Rigaud, & Sorci, 2009). The lower PO activity found in invading wasps (Wilson‐Rich & Starks, 2010) and gammarids (Cornet et al., 2010) can be analogous to the reduced investment in costly inflammatory response of vertebrates. However, none of these studies reports an increase in cellular immunity, thereby limiting a thorough examination of the EICA‐refined hypothesis.

To conclude, more than 75% of the studies reviewed here detect significant differences between immune responses of invasive and native species, or of invaders sampled along invasion routes. However, there is also a strong variability in the patterns observed that does not permit drawing general conclusions about the EICA hypotheses.

## 3 Overlooked and neglected aspects of the immune defences–bioinvasion relationships

The lack of general patterns with regard to EICA hypotheses may result from the relatively low number of studies found and their diversity in terms of methodologies (Table 1). Additionally, several major points including experimental designs, immune costs and infection data, for example, might be overlooked or neglected, although they are essential to the interpretation of the results. They are outlined below.

### 3.1 Experimental designs

Experimental/technical constraints are inherent in most, if not all studies. However, choosing relevant and appropriate designs to investigate whether changes in immune defences promote invasion success is not trivial, and may exert considerable influence on what can ultimately be concluded (van Kleunen, Dawson, Schlaepfer, Jeschke, & Fischer, 2010). Interspecific comparisons offer an interesting starting context to investigate whether differential life histories are associated with invasion ability; however, they come with a series of limitations. First, contrasted immune responses (constitutive or induced) may reflect interspecific differences in immune architecture only, and be unrelated to invasion success. Indeed, immune strategies and the strength of immune responses have been shown to vary significantly between closely related species (Buehler, Piersma, & Tieleman, 2008; Lee & Klasing, 2004; Martin, Weil, & Nelson, 2007; Mendes, Piersma, Hasselquist, Matson, & Ricklefs, 2006). Second, attention is rarely paid to the geographic location of the samples along the invasion gradient in interspecific comparisons (but see, Diagne et al., in revision). Immune alterations promoting invasion may be transient, and rapidly blurred by eco‐evolutionary changes following establishment (Sakai et al., 2001). Whether invaders come from anciently or recently established populations can also significantly impact the interpretation of results. Hence, we suggest that interspecific approaches should ideally include replicates of noninvasive and invasive congeners sampled at various points of the colonized area, as well as samples of invasive species from their native ranges (Monzon‐Arguello et al., 2014).

Studies based on “ongoing expansion range” designs are now becoming more frequent, suggesting that researchers have realized the potential and importance of this design to unravel the role of immune defences. This approach has the advantage of reflecting a spatial and temporal continuum in the invasion process; however, it requires substantial knowledge of the invasion routes and how the expansion range progressed to understand the evolutionary history of the species (Miura, 2007). All studies listed in Table 1 that focus on the “ongoing expansion range” design are based on such information. But successful introduction and establishment of invading species may partly result from stochastic processes related to population bottlenecks (Sakai et al., 2001; White & Perkins, 2012). As such, contrasted population dynamics and parasitic pressure may differ between invasion routes of a given species, as evidenced in Australian cane toad studies (Lettoof, Greenlees, Stockwell, & Shine, 2013; Urban, Phillips, Skelly, & Shine, 2008). Comparative analyses between invasion routes might be useful to demonstrate the consequences of stochastic and selective processes on immunity changes. Hence, in addition to both spatial and temporal surveys investigating where and when immune changes arise, an optimal study design should include population replicates, or compare several invasion routes (White et al., 2013).

### 3.2 Estimation of immune investment

A central issue in eco‐immunological studies concerns the assessment of immunocompetence through accurate estimates of immune responses (Adamo, 2004; Saks, Karu, Ots, & Hõrak, 2006). The majority of eco‐immunologists agree that the complexity of the immune system cannot be meaningfully captured using the measure of a single effector (Viney, Riley, & Buchanan, 2005). General and standard measures of immunocompetence, such as blood cell count or PHA challenge, may prevent detection of a specific immune pathway that is involved in the success of an invasion. Multifaceted immune responses are particularly relevant for testing the EICA‐refined hypothesis assumptions. These require the concomitant assay of effectors involved in different immune pathways that are assumed to have contrasted costs. However, such multiple immune approaches may be hindered by the number of measurements that can be taken on a single individual, which is often limited by the volume of blood or tissue that can be reasonably collected, especially for subjects of small body size. Given the immune system complexity, the assessment of immune parameters may reveal negative or positive correlations, as well as no pattern of association (Forsman, Vogel, Sakaluk, Grindstaff, & Thompson, 2008; Matson, Cohen, Klasing, Ricklefs, & Scheuerlein, 2006; Palacios, Cunnick, Winkler, & Vleck, 2012; Versteegh, Schwabl, Jaquier, & Tieleman, 2012). These findings are likely to vary depending on genetic, physiological or immunological constraints (Ardia, Parmentier, & Vogel, 2011). Future work should focus on tools allowing the investigation of as broad a range of immune effectors as is possible, depending on limitations imposed by blood or tissue sampling restrictions. In this context, the advent of immunomics—the study of the immune system regulation and responses to pathogens using genomewide approaches (Robertson, Bradley, & MacColl, 2016)—is highly promising. Few genome scan studies have yet been developed to examine the role that adaptive processes may play in the course of biological invasions (Quéméré et al., 2015; White et al., 2013). The use of transcriptomic approaches or high‐throughput gene expression measures targeting immune genes could also be interesting, as they have successfully been done in a variety of eco‐immunological studies, albeit not in an invasion context (Sevane, CaÒon, Gil, & Dunner, 2015).

### 3.3 Immunopathological costs and tolerance

The fitness cost associated with parasitic infections may actually not reflect the strength of the pathogenic pressure (Lippens, Guivier, Faivre, & Sorci, 2016). Such discrepancies between the strength of the pathogenic pressure and the actual underlying cost may involve regulatory mechanisms of immune activities that prevent immunopathologies. Tolerance is the set of strategies that limit the damage caused by a given parasite burden, including those that participate in regulating immunopathologies (Ayres & Schneider, 2008; Medzhitov, Schneider, & Soares, 2012; Råberg, Graham, & Read, 2009).

White and Perkins (2012) have suggested that host tolerance could favour invasion by mitigating fitness effects of infections and sickness behaviours associated with resistance in invaders. As tolerance is expected to increase parasite prevalence and lower virulence (Miller, White, & Boots, 2006; Roy & Kirchner, 2000; Vale, Wilson, Best, Boots, & Little, 2011), tolerance can favour the amplification of native parasite epidemiological cycles and increase prevalence in native hosts (spillback, Kelly et al., 2009). Tolerance can also foster the success of introduced species via spillover (Strauss, White, & Boots, 2012) and apparent competition mediated by introduced parasites on native hosts (Martin, Hopkins, et al. 2010). These potential relationships between tolerance and invasion have not yet been investigated although such research would clearly offer new perspectives. We advocate for field and experimental work dedicated to the evaluation of tolerance in invasive and native species, in both their native and invasion front populations. Capture–mark–recapture surveys of wild animals with varying levels of parasite burdens (Hayward et al., 2014) or analysing reaction norms of host immunity, host fitness and parasite burdens across environmental gradients in controlled conditions (Lippens et al., 2016) could be informative approaches to measure tolerance. Focusing on how tolerance mechanisms may trade‐off with immune resistance should in turn emphasize the role of parasites in invasion outcomes.

### 3.4 Assessment of infection risks and parasitological monitoring at a community scale

The EICA and EICA‐refined hypotheses assume changes in the selective pressures exerted by parasites on host species between the sites of introduction and the expansion ranges. However, such information is often lacking from studies investigating the immunity–invasion relationship (Table 1).

Data on parasite communities and infection risks (the combination of virulence and prevalence reflecting the strength of parasite‐mediated selection) in native and invaded areas are essential to interpret the immunological patterns observed in natural populations, or during immune challenge experimentations (Biard, Monceau, Motreuil, & Moreau, 2015). For instance, integrating a parasite perspective into the bioinvasion‐immunity framework may allow an understanding of the causal relationships between parasite exposure, infection and immunity. The loss of parasites by invasive hosts while expanding their range (“enemy release hypothesis”) has received some support in the literature, especially for studies comparing invasive populations in their native and introduced ranges (Colautti et al., 2004; Heger & Jeschke, 2014; Torchin, Lafferty, Dobson, McKenzie, & Kuris, 2003). Although this process is at the core of the EICA hypothesis, its consequences on host immune defences have to be carefully tested. First, parasite loss may exist with no further effect on host immunity or fitness (Colautti et al., 2004). Second, the immune alteration could be a function of the degree of parasite specialization. Some evidence suggests that specialist parasites are more prone to be lost by their host than generalist ones (Ewen et al., 2012; Heger & Jeschke, 2014). As these specialist parasites elicit, among other things, antibody‐mediated responses, invasive hosts that are no more in contact with them should take advantage of downregulating these adaptive responses. This may explain the lower genetic/functional diversity observed at *Mhc* genes in invasive populations when genetic drift alone cannot explain this pattern (Monzon‐Arguello et al., 2014; Quéméré et al., 2015). The loss of coevolved parasites during invasions may, however, come at a cost if the lack of exposure to immunosuppressive “old friends” substantially enhances the risk of suffering from immune disorders (Rook, 2013; Sorci, Cornet, & Faivre, 2013a,b). Having data on parasite communities and tolerance in invasive populations would help in evaluating whether the absence of coevolved parasites may alter the cost/benefit ratio of immune defences.

In numerous cases, invasive hosts do not experience parasite release, but rather changes in infection risks (Colautti et al., 2004). Introduced individuals often tend to acquire generalist parasites from the local fauna of their new range (Kelly et al., 2009). Unfortunately, infection risk related to local parasite faunas is far from being systematically studied in invasive species (but see Lettoof et al., 2013; Marsot et al., 2013; O'Brien et al., 2011). This has further consequences on immune alterations. The maintenance of high levels of costly immune responses (e.g. inflammation) in natural populations at the invasion front (Diagne et al., in revision; Martin et al., 2014; Quéméré et al., 2015) could indicate that invaders are exposed to high pathogenic pressure, although not necessarily infected. Alternatively, maintaining an ability to mount an acute phase response could be essential to survive in any environment, and explain why this defence is not traded‐off (Hegemann, Matson, Versteegh, & Tieleman, 2012). Considering the invasive hosts, it is worth noting that newly acquired, local parasites might elicit equivalent responses to those elicited by lost parasites, and explain why the EICA‐refined hypothesis is not supported in some cases.

Studying the concomitant evolution of host immunity and pathogen virulence during bioinvasion will help understand parasite transmission and epidemiology in introduced and native species of invaded ranges. How parasite traits change with expansion range (apart from a differential parasite prevalence/abundance) is another question that has received little interest so far. Changes in host immune investment may have hidden consequences on parasite infectivity and virulence (Cornet, Bichet, Larcombe, Faivre, & Sorci, 2014; Sorci et al., 2013a,b). Yet, pathogens could become more virulent as they spread into newly colonized populations and/or undergo changes to adapt to the new ecological conditions, as shown in a recent study on nematodes in invasive cane toads (Kelehear, Brown, & Shine, 2012). Given the intimate interplay between hosts and parasites in reciprocally driving parasite virulence and level of host immune investment (Mackinnon & Read, 2003, 2004), studying changes in parasite traits (e.g. transmission mode, virulence) along an invasion gradient may help better interpret the factors that drive variation in host immunity during bioinvasions.

## 4 Perspectives: New questions to address

### 4.1 What is the timing of immune changes?

Temporal surveys of host immune investment within invasion front populations have not been reported yet. Such studies would yield important information on the timing of immune changes associated with invasion. It is indeed important to decipher whether immune strategies favouring invasion success are a consequence of pre‐adaptation or the result of adaptation to the new ecological conditions encountered on the invasion front. For instance, the level of T‐cell‐mediated immune response in birds could be a prerequisite to invasion success rather than a consequence of it, as it was found to correlate positively with the success of establishment of introduced populations (Moller & Cassey, 2004). This conclusion needs to be generalized, and we advocate long‐term temporal surveys which would enable analysis of the processes of adaptation underlying invasion, including the role of immune changes and parasite‐mediated selection. In this context, Thomaz et al. (2012) proposed a conceptual framework based on a combined space‐for‐time and temporal monitoring of, respectively, invaded versus noninvaded sites, and pre‐invasion versus postinvasion sites. They advocate for the need to consider “negative controls” in these surveys. As such, the real‐time approach provides pre‐invasion data that enable better interpretation of invaded versus noninvaded site comparisons. The space‐for‐time approach in turn provides noninvaded data that can be used as negative controls when comparing pre‐ and postinvasion sites. This conceptual framework should limit the misinterpretation of results obtained during temporal or spatial approaches only as it separates the factors influencing invasion success (pre‐invasion state) from the impacts derived from invasion (postinvasion state). Hence, it suggests interesting practical alternatives for designing future eco‐immunological studies in the context of bioinvasions.

### 4.2 What are the causal mechanisms of immune changes?

The timing of immune changes raises a challenging question regarding the causal mechanisms underlying this phenotypic evolution during biological invasions. An altered immune response of invaders between source and invasion front populations may stem from different, nonexclusive mechanisms: adaptive phenotypic plasticity of individuals, selective evolutionary changes, and their interplay (Sakai et al., 2001).

A major part of immune variation relies on differences in genetic background that concern both specific recognition and immune regulation (Cotter & Wilson, 2002; de Craen et al., 2005; Frank, 2002; Graham et al., 2010; Kilpimaa, Van de Casteele, Jokinen, Mappes, & Alatalo, 2005; Lazzaro, Sceurman, & Clark, 2004). Significant levels of additive genetic variation in immune traits have been reported in invertebrates (Cotter & Wilson, 2002) and vertebrates (Graham et al., 2010), with levels of narrow sense heritability (the proportion of variation in phenotype due to genetic variation) ranging from moderate to high (reviewed in Ardia et al., 2011). Therefore, natural selection may be one of the key processes by which invaders become adapted to their new environment on the invasion front after few generations. Under this hypothesis, a lag phase is expected between the introduction of invaders on the invasion front and the establishment/range expansion. It could at least partly correspond to the time needed for pre‐adapted genotypes to increase in frequency in the population, or for adaptation to evolve in these established populations through mutations or genetic admixture due to multiple introductions (Keller & Taylor, 2008; Sakai et al., 2001). As advocated above, longitudinal surveys of an invasion front and sites just beyond are therefore of key importance to examine whether/how selection contributes to invasion success. It is important to keep in mind when analysing these selective forces in natural populations that nonadaptive spatial patterns may also be observed on the invasion front (reviewed in Charbonnel & Cosson, 2012). In particular, range expansion associated with intense genetic drift and limited dispersal may lead to genetic surfing (Excoffier & Ray, 2008), that is the propagation of any alleles by the invasion front of the invasion. As such, even deleterious mutations can reach high frequencies on the invasion front, leading to maladaptation persisting and being propagated over the course of the expansion. Such false‐positive signals of selection may be emphasized by analysing “replicates” of populations or of invasion routes, and by comparing phenotypic changes with null expectations.

Immune changes may also occur through phenotypic plasticity, that is the ability of one genotype to express different phenotypes across environmental contexts. This causal mechanism has been widely reported using experimental works modifying environmental conditions (resource quality and availability, cross‐fostering) or individual surveys throughout their life (immunosenescence, seasonal variation), in both vertebrates and invertebrates (Schmid‐Hempel, 2003; Schulenburg, Kurtz, Moret, & Siva‐Jothy, 2009). Whether plasticity of immune traits may facilitate or even speed up the process of adaptive evolution on the invasion front remains a challenging question. On the one hand, phenotypic plasticity may constrain or slow the rate of adaptive evolution by resulting in mean phenotypes being highly different from the optimum required on the invasion front (nonadaptive plasticity), and by shielding genotypes from the effects of selection (Ghalambor, McKay, Carroll, & Reznick, 2007). But on the other hand, phenotypic plasticity can be adaptive when leading to phenotypes that are close to the optimum favoured by selection in this new environment. It can even lead to an adaptive genetic response with the genetic fixation of a favourable phenotype through phenotypic canalization and genetic assimilation/accommodation (Ghalambor et al., 2007; West‐Eberhard, 2003).

From this perspective, it remains important to disentangle the relative, but nonexclusive, roles of genetic *versus* plastic changes from two points of view (i.e. the mean phenotype and the reaction norm), and during the different phases of the invasion process. For example, it would be interesting to test whether the phase lag between population establishment and expansion is made possible by adaptive phenotypic plasticity (Ghalambor et al., 2007), or to analyse whether phenotypic plasticity has evolved between source and invading populations. These questions can be addressed using common garden experiments where individuals collected in the field (in native and invaded areas) are reared, mated and bred in laboratory conditions with constant environmental conditions. If differences in immune defences between native and invaded areas are mostly resulting from plastic adjustments to environments, they should disappear in the following generations reared under similar laboratory conditions. On the contrary, the maintenance of differences in immune responses would suggest that the immune system has evolutionarily changed in response to invasion history. Such an approach has been used on the Australian cane toad system by Brown et al. (2015), who showed that compared with conspecifics from anciently colonized areas, cane toads whose parents originated close to the invasion front had higher innate immune responses (neutrophil concentration, bacteria‐killing activity and phagocytosis). Here, invasion seems to have resulted in rapid genetically based shifts in immune defences.

### 4.3 Evolution of dispersal and its immune consequences

Up to now, parasites have been at the core of hypotheses linking immunity and invasion success. A better understanding of these links would benefit from the consideration that immunity is also linked to other traits experiencing changes during the invasion process, such as dispersal (Llewellyn et al., 2012). The evolution of higher dispersal rates at the invasion front (Shine, Brown, & Phillips, 2011) has been described in many animals, including cane toads (Phillips, Brown, Webb, & Shine, 2006) and butterflies (Hughes, Dytham, & Hill, 2007). Several hypotheses have been proposed to explain how this evolution may explain immune changes between source and invasion front populations. They rely on the links established between dispersal and sexual hormones (Bowler & Benton, 2005), or between dispersal and stress levels (Segerstrom, 2007). They are also built on the existence of energetic (Llewellyn et al., 2012) or evolutionary (Snoeijs, Van de Casteele, Adriaensen, Matthysen, & Eens, 2004) trade‐offs between dispersal and immunity.

### 4.4 Exploring the links between age‐dependent immunity and adaptation on invasion front

Among the wide array of immune response characteristics, immune costs and specificity have been largely invoked to propose potential adaptive immune changes favouring invasion success. Other potential features that could be interesting to investigate include age‐dependent immunity. Studies of variation in immune responses during invasion have focused on adults only, although immunity strongly changes with age, both in intensity and in the relative importance of different immune pathways (e.g. in wild vertebrates, Cichon, Sendecka, & Gustafsson, 2003; Nussey, Watt, Pilkington, Zamoyska, & McNeilly, 2012; Palacios, Cunnick, Winkler, & Vleck, 2007; Ujvari & Madsen, 2011). The new environment encountered on the invasion front could, therefore, have varying effects on different age classes. A recent model developed by Cotto and Ronce (2014) showed that adaptation to new environments (e.g. during colonization) is faster when environmental changes mostly affect young individuals. In other words, maladaptation following environmental changes is higher for traits expressed late in life due to their slower response to selection. The age structure of introduced populations, as well as variation in the strength of environmental selection on age‐specific immune phenotypes on the invasion front, could be major parameters driving the phase lag before invaders adapt to these new conditions.

From this perspective, we propose that long‐term spatiotemporal surveys of biological invasions should take into account the age structure of introduced populations, and examine how this might accelerate or impede the establishment and expansion of these populations. Focusing on age‐specific immunity, we might further analyse whether the new environment encountered on the invasion front has different impacts on age‐dependent immune traits. In vertebrates, juveniles and younger individuals rely more on innate immune defences and maternally derived immunoglobulins than adaptive immunity, which develops later and takes more time to become fully functional (Boulinier & Staszewski, 2008; Garnier et al., 2012; Palacios, Cunnick, Vleck, & Vleck, 2009). It could, therefore, be interesting to follow adaptation of these three different immune pathways on the invasion front, and to measure selection acting on these age‐specific traits. Introduced populations facing strong environmental selection on juvenile immune traits should exhibit lower phase lag before expansion than populations facing strong selection on adaptive immunity.

### 4.5 A paradigm shift: moving EICA hypotheses from parasites to microbiota?

Gut microbiota is at the core of complex interactions with other functions, including immune responses (Kau, Ahern, Griffin, Goodman, & Gordon, 2011). Because the living host environment is an important factor impacting microbiota (reviewed in Rook, 2013), we propose the hypothesis that the new ecological conditions experienced during invasion, in particular resource diversity, quality and availability, can affect immune defences through changes of invaders’ microbiota composition. Several studies have reported modifications of feeding behaviour during invasions by analysing the evolution of morphological features adapted to the food resources available in the colonized environments (lizards: Van Kleeck, Chiaverano, & Holland, 2015; mice: Renaud et al., 2015). Direct evidence for change in gut microbiota associated with biological invasions was only recently described by Minard et al. (2015). They showed that the gut microbiota of tiger mosquitoes was less diverse and more homogeneous in French invasive populations than in Vietnamese autochthonous populations. In vertebrates, such a decrease in gut microbial diversity may lead to the dysregulation of the inflammatory response (references with regard to men and mice in Rook, 2013). We therefore suggest that exploring more deeply the changes in diversity and composition of gut microbiota during biological invasions, and its links with immune changes and invasion success, is an exciting and potentially rewarding area of research for the future.

## 5 Conclusions

This perspective aims to promote a deeper understanding of the reciprocal links between immunity and the success of biological invasion. Although the diversity of studies reviewed here has undoubtedly helped biologists solve important issues, it has also come with a new set of questions. Studying the changes of immune defences associated with species expansion has proven more challenging than initially thought. Such work requires an integrated view of physiological, immunological, parasitological, ecological and evolutionary variables. Future work should aim to disentangle the timing and relative roles of selection and plasticity in invasion success. They should also examine how shifts in immune investment and other life‐history traits modify host resistance and tolerance to pathogens, and how these shifts ultimately impact parasite communities and virulence. These studies could be essential given the closely related issues of biological invasion and (re)emergence of infectious diseases (Sorci et al., 2013a,b; Brock, Murdock, & Martin, 2014).
